# Constellation: a tool for rapid, automated phenotype assignment of a highly polymorphic pharmacogene, *CYP2D6*, from whole-genome sequences

**DOI:** 10.1038/npjgenmed.2015.7

**Published:** 2016-01-13

**Authors:** Greyson P Twist, Andrea Gaedigk, Neil A Miller, Emily G Farrow, Laurel K Willig, Darrell L Dinwiddie, Josh E Petrikin, Sarah E Soden, Suzanne Herd, Margaret Gibson, Julie A Cakici, Amanda K Riffel, J Steven Leeder, Deendayal Dinakarpandian, Stephen F Kingsmore

**Affiliations:** 1 Center for Pediatric Genomic Medicine, Children’s Mercy-Kansas City, Kansas City, MO, USA; 2 Department of Computer Science and Electrical Engineering, School of Computing and Engineering, University of Missouri-Kansas City, Kansas City, MO, USA; 3 Division of Clinical Pharmacology & Therapeutic Innovation, Children’s Mercy-Kansas City, Kansas City, MO, USA; 4 Department of Pediatrics, Children’s Mercy-Kansas City, Kansas City, MO, USA; 5 School of Medicine, University of Missouri-Kansas City, Kansas City, MO, USA; 6 Deparment of Pediatrics and Clinical Translational Science Center, University of New Mexico Health Science Center, Albuquerque, NM, USA; 7 Department of Pathology, Children’s Mercy-Kansas City, Kansas City, MO, USA

## Abstract

An important component of precision medicine—the use of whole-genome sequencing (WGS) to guide lifelong healthcare—is electronic decision support to inform drug choice and dosing. To achieve this, automated identification of genetic variation in genes involved in drug absorption, distribution, metabolism, excretion and response (ADMER) is required. *CYP2D6* is a major enzyme for drug bioactivation and elimination. CYP2D6 activity is predominantly governed by genetic variation; however, it is technically arduous to haplotype. Not only is the nucleotide sequence of *CYP2D6* highly polymorphic, but the locus also features diverse structural variations, including gene deletion, duplication, multiplication events and rearrangements with the nonfunctional, neighbouring *CYP2D7* and *CYP2D8* genes. We developed Constellation, a probabilistic scoring system, enabling automated ascertainment of CYP2D6 activity scores from 2×100 paired-end WGS. The consensus reference method included TaqMan genotyping assays, quantitative copy-number variation determination and Sanger sequencing. When compared with the consensus reference Constellation had an analytic sensitivity of 97% (59 of 61 diplotypes) and analytic specificity of 95% (116 of 122 haplotypes). All extreme phenotypes, i.e., poor and ultrarapid metabolisers were accurately identified by Constellation. Constellation is anticipated to be extensible to functional variation in all ADMER genes, and to be performed at marginal incremental financial and computational costs in the setting of diagnostic WGS.

## INTRODUCTION

Cytochrome P450 family 2, subfamily D, polypeptide 6, CYP2D6, is one of the most important enzymes of bioactivation or elimination of endogenous and exogenous biochemicals. Specifically, CYP2D6 contributes to hepatic metabolism of ~25% of drugs in clinical use, including many antidepressants, antipsychotics, opioids, antiemetics, anti-arrhythmics, β-blockers, cancer chemotherapeutics and drugs of abuse.^1,2^ The enzymatic activity of CYP2D6 varies widely among individuals, based both on level of expression and on functional genetic variations (alleles), resulting in significant clinical consequences for drug metabolism and individual risk of adverse events or drug efficacy (www.cypalleles.ki.se/^3^ and www.pharmgkb.org/).

Responding to increased awareness of individual variation in drug metabolism, information regarding CYP2D6 is now included in the FDA-approved or European Medicines Agency (EMA)-labels of 51 drugs. The conventional phenotypic classification system defines predicted CYP2D6 activity as poor, intermediate, extensive and ultrarapid metabolisers. An individual’s phenotype may profoundly impact drug efficacy and potential for adverse reactions at standard medication dose. For example, mothers categorised as CYP2D6 ultrarapid metabolisers (OMIM#608902), and taking normal doses of codeine for post-partum pain relief, while breast feeding, convert codeine to morphine quickly resulting in high levels of morphine in breast milk that can lead to death in nursing infants.^4^ Likewise, children who are ultrarapid CYP2D6 metabolisers and taking codeine following adenotonsillectomy are at risk for toxicity and death.^5^ Exposure to the commonly used selective serotonin reuptake inhibitor fluoxetine is approximately fourfold higher in CYP2D6 poor metabolisers compared with adults with normal function.^6,7^ Furthermore, numerous drugs inhibit CYP2D6 activity, confounding the effects of genetic variation and resulting in adverse drug–drug interactions in situations of polypharmacy.^8^ As a result, expert consensus guidelines are being published to improve the safe and effective use of several CYP2D6 substrates.^9–12^

**Figure 1:**
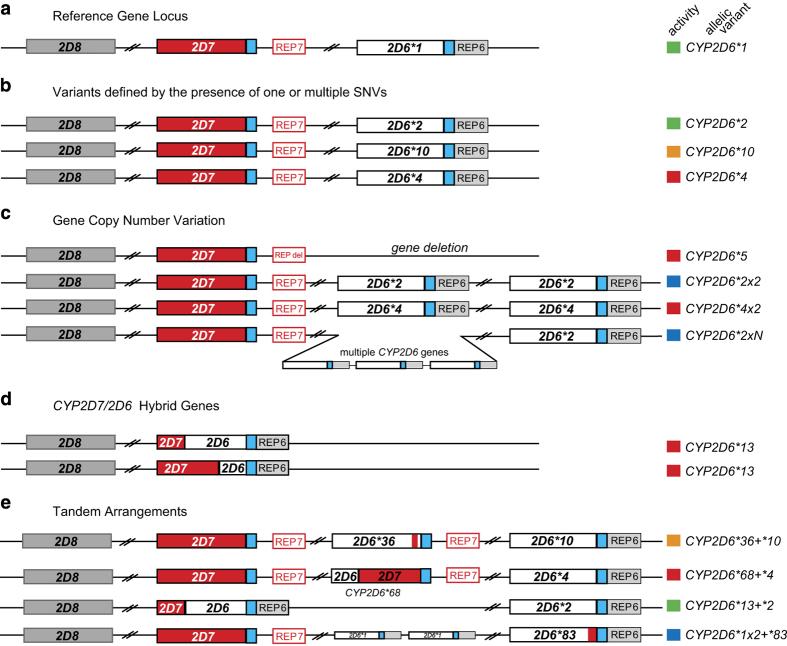
Graphical overview of the highly polymorphic *CYP2D6/2D7/2D8* locus. (**a**) The reference Chr 22 locus comprising the CYP2D6*1 haplotype (white) and two non-functional paralogs, *CYP2D7* (red) and *CYP2D8* (grey). Note that the locus is on the minus strand and is shown in reverse. REP6 and REP7 are paralogous, *Alu*-containing, 600-bp repetitive segments found downstream *of CYP2D6* and *CYP2D7*, respectively. The blue boxes indicate identical unique sequences downstream of *CYP2D6* and *CYP2D7* which are separated from REP7 by 1.6-kb in the latter. (**b**) Three *CYP2D6* haplotypes, CYP2D6*2, CYP2D6*10 and CYP2D6*4. The CYP2D6 activity conveyed by these haplotypes is indicated by colour-coded boxes (red, non-functional variant; orange, decreased activity; green, fully functional reference activity; blue, increased activity). (**c**) The most common *CYP2D6* copy-number variations. CYP2D6*5 is characterised by a deletion of the entire *CYP2D6* gene and fusion of REP6 and REP7 (REP-del). Duplication haplotypes have two or more *CYP2D6* copies, as exemplified by CYP2D6*2x2 (ultrarapid metaboliser) and CYP2D6*4x2 (non-functional). Less common are copy-number variants with three or more copies. (**d**) Hybrid genes composed of *CYP2D7* and *CYP2D6* fusion products that result from unequal recombination. A number of hybrid genes with a variety of switch regions have been described and are consolidated as the CYP2D6*13 haplotype. (**e**) Four tandem arrangements, featuring two or more, non-identical copies of *CYP2D6*.

The region of human chromosome 22 to which *CYP2D6* maps is highly polymorphic. In addition to *CYP2D6*, the 37-kb region contains a homologous, nonfunctional gene that arose through gene duplication (*CYP2D7),* and a pseudogene that arose through gene conversion (*CYP2D8)*.^13^ The *CYP2D* locus also contains GC-rich regions, and two, Alu-rich, 2.8-kb repeated regions *(REP6 and REP7)* that are substrates for a wide variety of common structural variations of *CYP2D6*, including copy-number variations (CNVs), gene conversions, rearrangements, and combinations thereof^14^ (Figure 1). Of the over 100 allelic variants (not counting subvariants) defined today by the Human Cytochrome P450 Nomenclature Committee (www.cypalleles.ki.se/^3^), many confer altered enzymatic activity. Given this complexity, the routine clinical determination of individual CYP2D6 activity by genetic analysis remains challenging,^15^ with a comprehensive analysis of the single-nucleotide variations, including insertions and deletions, CNVs, gene conversions and gene rearrangements characteristic of the *CYP2D6* locus requiring locus-specific amplification followed by a series of quantitative PCR reactions in addition to several long-PCR reactions and, occasionally, Sanger sequencing to unambiguously identify the specific combination of two haplotypes (diplotype) that is predictive of an individual’s CYP2D6 activity, a process that is both costly and labour intensive.

As discussed by Drögemöller *et al.* next-generation sequencing (NGS) is a powerful tool for variant detection, but a number of factors including high similarities to pseudogenes, GC content, repetitive or low-complexity sequences and a high degree of variation, may affect the analysis of *CYP* genes, in particular *CYP2D6*.^16^ As pointed out in their critical analysis, poor sequence coverage and poor mapping quality of reads qualified *CYP2D6* to be masked as ‘inaccessible genome’ in the 1,000 Genomes Project’. Improved NGS methods such as whole-genome sequencing (WGS) in conjunction with bioinformatic tools may, however, overcome these limitations.

WGS has successfully been applied for the molecular diagnosis of genetic diseases.^17–19^ The Illumina HiSeq X Ten, although not FDA-cleared, has the capacity to sequence ~18,000 human genomes per year to ~30-fold coverage at a sequencing cost of ~$1,000 per sample (www.nature.com/news/is-the-1-000-genome-for-real-1.14530) markedly changing the potential cost effectiveness of WGS in health-care applications. In a pediatric context, WGS is increasingly being used clinically, particularly in neurodevelopmental disorders, to diagnose suspected underlying genetic diseases,^20^ and it is not unreasonable to expect that at some point in the not too distant future, WGS data will be the rule rather than the exception. Critical to lifelong, individualised drug choice and dosing is the identification of genetic variation in genes critical to drug absorption, distribution, metabolism, excretion and response (ADMER) obtained at any point along the age continuum. In order to be of broad clinical use, scalable, automated methods are needed for imputation of function and/or activity of ADMER genes, with return of results to support clinical guidance for drug, dose and exposure for individual patients. At present about 100 ADMER genes are relevant for such guidance (http://www.fda.gov/drugs/scienceresearch/researchareas/pharmacogenetics/ucm083378.htm, https://www.pharmgkb.org/gene/PA128#tabview=tab0&subtab=32, and http://pharmaadme.org/), and of these, *CYP2D6* is the most technically difficult to diplotype. Although it may be somewhat premature to recommend WGS as a platform for routine pharmacogenomic testing, WGS data is becoming increasingly more common as a clinical diagnostic platform, and the pharmacogenome represents ‘secondary findings’ that may have direct applicability to the choice of medication as well as the most appropriate dose for an individual patient.^21,22^ Herein, we describe a system for scalable, automated derivation of diploid functional alleles from unphased WGS using *CYP2D6* as a specific example of its utility.

## RESULTS

### *In silico* modelling

**Figure 2:**
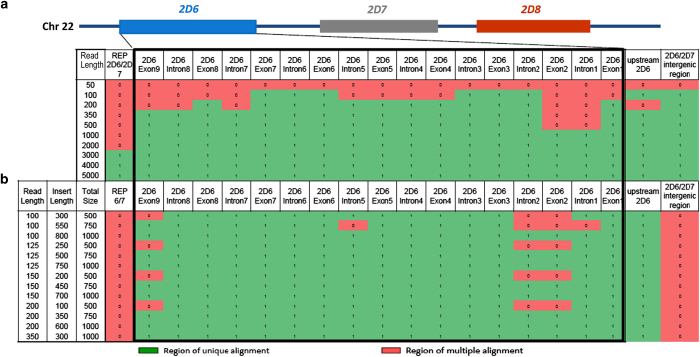
*In silico* modelling of the uniqueness of alignments of simulated short-read sequences to the region of Chromosome 22 containing *CYP2D6, CYP2D7* and *CYP2D8* (hg19, chr22:42,518,000-42,555,000). Simulated singleton reads (**a**) and paired-end reads (**b**) from 50 to 5,000-nt in length were generated from this region. For paired-end reads, insert lengths varied from 300 to 800-nt. Exons, introns and genomic features to which reads mapped uniquely with GSNAP are shown as green‘1’; regions to which reads did not map uniquely are shown as red ‘0’.

We assessed *in silico* whether short read sequences aligned correctly within the *CYP2D6* locus. Variant-free reads were tiled across the 37-kb CYP2D6*2 region at 5-nt spacing and aligned to the CYP2D6*2-containing reference genome (GRCh37) with the algorithm GSNAP (Figure 2).

No reads of any size or format misaligned, however, 20% of 100 nt singleton reads aligned ambiguously; Supplementary Figure 2A). This was expected based on the high sequence similarity between *CYP2D6* and *CYP2D7*. This ambiguity included *CYP2D6* exons required for the determination of functional haplotypes. *CYP2D6* exonic and intronic alignment ambiguity was unique at 1,000-nt, and across the entire locus at a read length of 3-kb. Using simulated standard sequencing parameters (paired 100-nt reads separated by 300 nt), *CYP2D6* exonic ambiguity was limited to exon 2 (Supplementary Figure 2B). Exonic and intronic alignment ambiguity resolved with 2×100-nt reads separated by 800-nt, 2×125-nt reads separated by 500-nt, or 2×200-nt reads separated by 350-nt. None of these, however, resolved the repetitive regions located upstream and downstream of the *CYP2D6* gene, or the *CYP2D7/CYP2D6* intergenic region. It should be noted that these models represent an ideal situation without sequencing errors or nucleotide variants.

### Probabilistic *CYP2D6* allele determination from WGS

**Figure 3:**
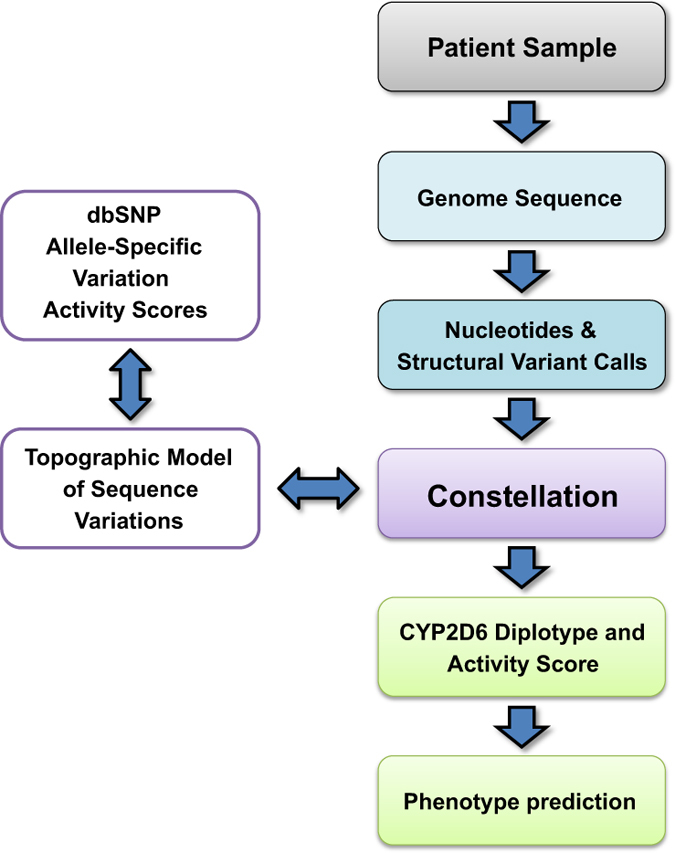
Flow diagram of the assignment of CYP2D6 phenotype inferred by WGS and Constellation. Whole-genome sequence data are mapped to the reference human genome and *CYP2D6* diplotypes called by Constellation. Predicted phenotype is determined by assigning an ‘activity score’ based on the individual diplotype.^8–10^

Having determined that the alignment to *CYP2D6* exons was largely unique with current read lengths (2×100 with 350-nt insert; Figure 2b and Supplementary Figure 2B simulation A), we developed Constellation (Figure 3), an algorithm to impute *CYP2D6* diplotypes from WGS. The development of this algorithm was driven by the need to phase the myriad of *CYP2D6* sequence variations into known (defined) haplotypes to maximise accuracy of genotype assignments. Global or local sequence alignment algorithms fail because of noise due both to sequencing errors and variants that are not represented in known/defined *CYP2D6* alleles. The latter is particularly crucial as some *CYP2D6* allele definitions are based on SNPs in exonic regions rather than complete haplotype sequences. Furthermore, there are no rigorous commonly accepted scoring algorithms such that it is difficult to recognise the correct solution among several possible candidates. Thus, the problem is akin to *de novo* peptide sequencing from tandem mass spectrometry in the presence of false positives and false negatives.^23^ A probabilistic scoring system was developed to determine the most likely diplotype match to the WGS-derived .vcf file (*V*_t_) of a test sample, *t*, based on prior computation of all theoretical haplotypes and corresponding functional alleles (as defined by the Human P450 Nomenclature Committee). For 119 *CYP2D6* haplotypes (Supplementary Table 3) the defining variant set was determined. Among those are 24 haplotypes with normal, 12 with decreased and 34 with no function; the *in vivo* function for the remaining 49 alleles is unknown or uncertain. The complete set of 7,140 possible diplotypes (119 choose 2 plus 119) was generated by combining the variant sets for each pair of haplotypes. For WGS of test sample *t*, Constellation retrieved the position and zygosity of each variant in the .vcf file, *V*_t_. that was compared with each possible diplotype *D*_1-7140_. For a diplotype *D*_a_ and *V*_t_, *X* variants were common, *Y* variants were in *V*_t_ only, and *Z* variants were found in the *D*_a_ only [*X*=(*V*_t_∩*D*_a_), *Y*=(*V*_t_−*D*_a_), and *Z*=(*D*_a_−*V*_t_)]. A variant location that was homozygous in *V*_t_ but heterozygous in the *D*_a_ set resulted in *X+*1 and *Z*+1 score adjustments. A Jaccard similarity coefficient^24^ could potentially be used to represent the probability of match *P*_1-7140_ of *V*_t_ for each *D*_a_. However, this assumes variant calling is error free.

To adjust for variant call errors, the scores were adjusted by the sensitivity (sens) and specificity (spec) of WGS variant calling. Assuming independence of variant calls, the score for each variant was reported as a likelihood ratio. For instance, a reported variant (type X) that matched a candidate diplotype was scored as P(Predicted|Present)/P(Predicted|Absent)=Sensitivity/(1−Specificity), type *Y* scored as P(Predicted|Absent)/P(Predicted/Present)=(1−Specificity)/Sensitivity, and type *Z* scored as P(Not Predicted|Present)/P(Not Predicted|Absent)=(1−Sensitivity)/Specificity. Thus, *X* was adjusted by *A*=[sens/(1−spec)], *Y* adjusted by *B*=[(1−sens)/spec], and *Z* adjusted by *C*=[(1−spec)/sens]. The overall score was the product of likelihood ratios of a diplotype sample set match [score=(*A* ^*x*^)*(*B* ^*y*^)*(*C* ^*z*^)]. Resultant diplotypes were returned in a reverse sorted list with the highest index, max(*P*), reported to the output file. The CYP2D6 activity corresponding to the highest scoring diplotype was reported.

To evaluate the ability to resolve diplotypes, all 7,140 defined diplotypes were simulated and analyzed using Constellation. Of the 7,140 possible diplotypes, 7,130 were correctly identified. For 10 cases, the correct diplotype was returned along with one alternative diplotype call. In six of those instances Constellation was not able to discriminate suballeles. For example, the CYP2D6*1A/*2G test case was called as *1A/*2G or *1D/*2C. In the remaining four instances, diplotypes could not unequivocally be resolved due to partial alternative suballeles matches. For example, the test case CYP2D6*2D/*6B was called as *2D/*6B or *6C/*34 and the test case CYP2D6*6C/*34 was called *2D/*6B or *6C/*34. For additional results see Supplementary Materials.

### CNV calls by Constellation

Detection of CNV across the *CYP2D6* locus proved challenging due to the prior described hybrid gene rearrangements, full gene deletions and full gene duplications present in the locus. A depth of coverage analysis was employed to evaluate the sample’s Bam file for variations in read coverage. To detect CYP2D6*5 characterised by a deletion of the entire gene, two separate sentinel regions were used, a control region *C*_R_ (GRCh37, chr22 42528247–42531055) and a variable target region inside the gene locus *C*_T_ (GRCh37, chr22 42520084–42521067). A ratio of *C*_T_/*C*_R_ between 0.25 and 0.4 was observed for samples carrying the CYP2D6*5 deletion allele. We did not observe any CYP2D6*5/*5 subjects making it difficult to establish a cutoff value for homozygous subjects. To adjust for small local variation in coverage the average depth across a region was used as the depth of coverage value in the calculation. As a deletion event results in about half the number of reads and also reduces the signal noise of depth of coverage across the locus, deletion events were readily detectable.

Gene duplication or multiplication and rearrangement events that formed *CYP2D6/7* and *CYP2D7/6* hybrid genes were more challenging to detect. The presence of a gene duplication is expected to result in a signal increase by a third, as well as an increase in overall noise in the alignment depth of coverage signal. Both event types, duplications/multiplications and gene hybrids were tested for by using multiple sentinel regions, i.e., the control region *C*_T_ described above and exon *E*_1-6,8,9_ as the targets. Normal ranges were determined for each exon independently of each other; the exon 7 depth of coverage was too noisy and was therefore excluded from analysis. Ratios of *C*_T_*/E*_1-6,8,9_ served as an indicator for the presence of a CNV event (*E*_1_>2.4, *E*_2_>2.5, *E*_3_>2.75, *E*_4_>3.0, *E*_5_>3.0, *E*_6_>2.8, *E*_8_>3.0, *E*_9_>3.0). Samples with a duplication had all exons outside the normal range.

### Analytic performance of Constellation

To evaluate the performance of Constellation, *CYP2D6* alleles were ascertained in 61 samples by manual integration of results obtained by quantitative copy-number assessment, a panel of TaqMan genotype assays, and Sanger sequencing of long-range genomic PCR products (the combination of these constitute the ‘consensus reference’) and probabilistic WGS analysis by Constellation (Table 1, Supplementary Table 2 and Supplementary Figure 3). The analytic sensitivity and specificity of WGS for nucleotide genotypes with the read alignment and variant calling methods employed was 98.78% and 99.99%, respectively; this was determined by comparing sample NA12878 with reference genotypes provided by the National Institute of Science and Technology.^25^ Formal *CYP2D6* allele definitions were converted to pseudohaplotypes, i.e., by a set of discontinuous variants, by reference to the human genome GRCh37.p13. The inheritance of all consensus reference method diplotypes in familial trios and tetrads followed rules of segregation.

The analytic sensitivity of Constellation was 98% (59 of 61 samples, Table 1). In the remaining two samples Constellation returned more than one possible diplotype. In addition, Constellation correctly detected copy-number gains (*n*=2) or losses (*n*=5) in seven samples. Constellation had two calls deviating from the consensus reference calls: sample CMH570 was called as CYP2D6*39/*95 rather than CYP2D6*1/*1 and CMH673 was called as CYP2D6*83/*35 instead of CYP2D6*1/*35. The CYP2D6*39/*95 miscall leads to an ‘unknown’ phenotype assignment (the activity of both alleles in ‘uncertain or unknown) while the consensus reference genotype predicts an activity score of 2 indicating normal activity. For the CYP2D6*83/*35 miscall Constellation and the consensus reference genotypes both predict extensive metaboliser phenotype.

Regarding the detection of structural variants, quantitative copy-number analysis indicated the presence of CYP2D6*68+*4 tandem arrangements in seven individuals. This structure (Figure 1) cannot be detected by TaqMan genotyping or Sanger sequencing, or consistently with Constellation. In all cases though, CYP2D6*68+*4 was correctly defaulted to CYP2D6*4, which accurately identified a no-function allele.

Each of the components of the consensus reference only detects certain aspects of *CYP2D6* variation. Results obtained for each component, i.e., copy-number number analysis, TaqMan genotyping and Sanger sequencing, are provided in Table 1.

### Concordance of CYP2D6 phenotype prediction

Assignment of correct activity is critical to transition from raw sequencing output to genome-informed drug guidance and precision medicine. Activity scores were assigned to the diplotypes obtained from each platform (TaqMan genotyping, Sanger sequencing and Constellation) and compared (Table 1). The activity of some *CYP2D*6 diplotypes is uncertain (function of one or both alleles is unknown at this time), hence it is not possible to predict activities for all of the experimentally defined diplotypes. The clinical sensitivity of Constellation was 93% (an activity score was assigned for 57 of 61 subjects) and that of the consensus reference method 98% (an activity score was assigned for 60 of 61 subjects). The clinical specificity of Constellation was 98% (56 of 57 Activity Scores (excluding no calls and unknowns) were concordant with the consensus reference). Importantly, all extreme phenotypes, i.e., poor and ultrarapid metabolisers were correctly identified with Constellation (Table 1).

### Novel *CYP2D6* haplotypes identified by WGS and Sanger sequencing

Fifteen nucleotide variants were identified by WGS and Sanger Sequencing that are not part of currently defined *CYP2D6* alleles (haplotypes; Table 1, Supplementary Figure 3). These SNPs define five subvariants of CYP2D6*1 (*var1*–*5*), two subvariants of CYP2D6*2 (*var1, 2*) and four subvariants of CYP2D6*4 (*var1*–*4*). One subvariant of CYP2D6*17 (var1 has previously been described^26^ (see Supplementary Information for additional details). Notably, rs267608274 (424C>T) was identified in two related subjects by Sanger (mother and child 2), but only called by NGS using GSNAP-GATK for child 2. This SNP was not identified by the variation caller in the mother owing to a low quality threshold. We also identified a CYP2D6*17 subvariant that is characterised by the lack of three SNPs (family 5, subject CMH438 in Supplementary Figure 3).

An additional 27 sequence variations were identified by WGS, but not Sanger sequencing (Supplementary Figure 3). The majority are within regions that are known to harbor *CYP2D7*-derived segments on certain *CYP2D6* haplotypes such as those accepted in the pharmacogenomics field as *CYP2D7* intron 1 and exon 9 conversions, or carry sequence variations matching *CYP2D7*. The most likely explanation for these false-positive calls is nonspecific read alignment. These regions were also identified by our *in-vitro* modelling to be the most challenging for accurate alignment of NGS (Figure 2 and Supplementary Figure 2).

## DISCUSSION

Herein, we have described Constellation, a computational method for automated derivation of diploid functional haplotypes from unphased WGS, and demonstrated the analytic and clinical utility of the method as applied to the challenge presented by *CYP2D6*.

There is a strong need for timely CYP2D6 activity information to guide the choice of medication within and between classes of drugs where therapeutic alternatives exist, and for selection of initial dose.^6,9–12,27^ The latter is especially important in pediatric practice, where FDA-labelled dosing guidance is often absent, efficacy is unproven and toxicity is concerning.

Pharmaceutical choice and initial dose selection is crucial in children with neurodevelopmental disabilities for whom CYP2D6 substrates, such as aripiprazole, atomoxetine, citalopram, fluoxetine, fluvoxamine and risperidone, are commonly prescribed.^27^ Children with developmental disabilities are uniquely vulnerable to the limitations of subjectively guided medication management, the mainstay of current practice, screening for side effects and assessment of target symptoms such as anxiety and irritability. Exome and genome sequencing of children with neurodevelopmental disabilities for aetiologic diagnosis is rapidly becoming standard of care in light of recent reports showing rates of diagnosis of single gene disorders of 31–47% in this population.^20,28,29^ For this group, automated return of actionable pharmacogenomic secondary findings in diagnostic WGS reports is highly desirable for implementation of precision pediatric neurology and psychiatry.^30^ As discussed below activity scores could be provided as potentially actionable, secondary findings in diagnostic WGS reports for a modest increment in cost. Although not included in the current American College of Medical Genetics guidelines, a panel of pharmacogenomic activity scores fits well with the more recent American Society of Human Genetics guidelines with respect to reporting of secondary findings in infants and children.^31^ Prospective studies of the clinical and cost effectiveness of WGS-based return of *CYP2D6* secondary findings in this population are warranted.

Specific pharmaceutical selection within a class is especially important when the therapeutic index is narrow,^32^ and in indications where biological responses take weeks or months to measure. This is exemplified by the selective serotonin reuptake inhibitors^33^ for young children, with poorly defined starting dose, compounded by parent comfort level and provider experience.^34^ Dose adjustments are based largely on parent and teacher impressions of medication tolerance and effect, requiring 4 weeks post initiation of treatment. Self-reports in pediatric populations may be absent or difficult to interpret. Individuals with alleles that increase CYP2D6 activity at standard starting dose result in lower than expected drug levels and risk treatment failure, not apparent clinically until at least one month into treatment. Conversely poor metabolisers may have toxicity at typical doses, resulting in risk of serotonin syndrome, or increased risk of known adverse reactions including suicidal ideation, activation and treatment-induced mania. For these reasons genotype-aided dosing is increasingly being recognised as important.^33^

Despite the central importance for clinical pharmacogenomics and precision medicine, no current ‘gold standard’ method exists for clinical determination of *CYP2D6* (or other pharmacogene) diplotypes and their translation into clinically actionable results.^15^ Regardless of the genotyping or sequencing methods used considerable knowledge regarding genome sequence nomenclature and conventions, *CYP2D6* haplotype (star allele) nomenclature, and *CYP2D6* haplotype—CYP2D6 phenotype relationships is required. Furthermore, mappings between these are not necessarily intuitive,^35^ one-to-one or fixed with respect to time, which may pose a barrier to the general adoption of interpretation of *CYP2D6* genetic results. Other computational methods such as Cypiriri have been developed to assess *CYP2D6* genotype from high-throughput sequence data.^36^ Although Cypiripi was evaluated on 71 simulated data sets, its validation was limited to a Coriell trio (NA12878, NA12877 and NA12882). Similar to Constellation, the CYP2D6*68+*4 allele was called as CYP2D6*4 missing the additional gene copy. The Cypiripi algorithm also heavily relies on locus-specific analysis techniques such as alignment to custom reference sequences and identification of common spurious variant calls from the *CYP2D7* pseudogene. Constellation is advantageous as this tool is a homogenous method that is rapid, scalable and has minimal incremental cost in the setting of a whole-genome sequence through its ability to use the VCF output from the primary alignment and variant detection pipeline without imposing an additional computational burden. This allows for the parallel processing of multiple loci with annotated nomenclature systems without requiring locus-specific reanalysis or any knowledge of related genes and/or pseudogenes. As Constellation adjusts haplotype scoring based on the sensitivity and specificity of the variant detection method being used, any improvement in variant calling in the primary analysis pipeline, either through improved read format or pipeline parameterisation, is immediately available to Constellation to improve locus resolution. Finally, Constellation has minimal requirements for expert domain knowledge for operation, as it performs the intermediate mapping, translation and inference steps.

Given the complexity of variation in *CYP2D6*, the variable quality of haplotype definitions, and broad types of variation seen in the samples, Constellation performed well by producing calls that allowed us to assign activity scores for 57 of the 61 subjects. We attribute the higher success rate obtained for the consensus reference (assignment of activity scores for 60 subjects) to the exhaustive testing that exceeded genotype testing routinely offered by commercial companies. Comparing a combination of TaqMan and CNV for example with Constellation, allelic variants with decreased or no function would have been missed in four subjects (Table 1).

Although the samples tested represented the diversity and complexity of *CYP2D6* nucleotide and structural variation, they did not include all possible haplotypes, especially rare alleles with structural variations. For routine clinical use, Constellation will require clinical-grade WGS as an input, additional software documentation and further validation studies to meet CLIA/CAP guidelines, performance in a CLIA/CAP laboratory, and official reporting of results by a qualified laboratory director.

Manual curation and confirmatory testing will likely be necessary for a small number of samples including those with ambiguous calls, or samples for which Constellation called extremely rare alleles or a combination of rare alleles. Cases with complex rearrangements and gene duplications may also require additional testing to unequivocally determine the number of functional gene copies. The number of subjects requiring additional testing is currently <5% and will likely decrease as the algorithm is further improved, e.g., more complete allele definitions become available and/or WGA read length/insert size allow for more accurate alignments. Cases such as CMH570 and CMH673 with CYP2D6*39/*95 and *35/*83 genotypes are examples that can be resolved by manual review of the WGA data and have the prospect of being accurately called as the algorithm is being improved over time. Nevertheless, it seems likely that CYP2D6 activity scores could provide potentially actionable, secondary findings in diagnostic WGS reports for a modest incremental cost, suggesting that cost effectiveness may be relatively easy to demonstrate.

There were four principal limitations to the performance of Constellation. Firstly, Constellation did not consistently detect CYP2D6*68+*4, a tandem arrangement featuring a hybrid *CYP2D6/CYP2D7* gene (*68) upstream of a nonfunctional CYP2D6*4 (Figure 1). Given the sequence similarity of *CYP2D6* and *CYP2D7*, this is extremely difficult to differentiate from an allele that carries CYP2D6*4 alone without complementary analyses such as our quantitative CNV assay^37^ or specific long-range genomic PCR (Supplementary Figure 1). However, because CYP2D6*68+*4 and CYP2D6*4 are both nonfunctional, discriminating them does not improve phenotype prediction. Further study is needed to assess whether Constellation can differentiate other tandem arrangement variations. Second, there are still haplotypes with unknown function impeding adoption by clinicians. In the current study this was observed in two of 61 subjects (3%). Third, GSNAP/GATK called a number of false-positive SNPs, which could tentatively interfere with Constellation calls. However, we also stress that our variant detection pipeline is parameterised in favour of sensitivity and to err on the false-positive rather than false-negative side. Increasing the variant detection stringency will likely decrease the number of false-positive calls. Last, not all *CYP2D6* variation has been cataloged and not all allele definitions that are listed by the *CYP2D6* Nomenclature web page are based on complete gene sequences. In this study we detected 13 suballelic variants of *CYP2D6* haplotypes (Supplementary Table 4 and Supplementary Figure 3) of which only one has been described in the literature^26^ (also see Supplementary Results). Of those, 12 carried novel SNPs and one (CYP2D7*17 var2) lacked three SNPs. Although these subvariants do not have diagnostic value to the best of our knowledge, updating Constellation with new information (novel haplotypes or additional information on known haplotypes) will improve accurate SNP phasing and haplotype/diplotype calling by the algorithm. Furthermore, as discussed by Fujikura *et al.*,^38^ NGS-based methods will discover novel, rare variations in *CYP* genes that elude commonly used SNV platforms over time and that this information will be critical to individualise treatment with drugs metabolised by CYPs. Of note, there is currently no central data base systematically capturing allelic variation for *CYP2D6* (or other pharmacogenes). This has two consequences for the performance of Constellation. First, mismatch errors can occur if the library of functionally relevant haplotypes and their defining variant sets is not comprehensive as exemplified in more detail in Supplementary Results. This may also have accounted for two Constellation ‘no calls’. Secondly, incomplete information raises concerns regarding readiness for routine clinical implementation.

To assess the utility of whole-exome sequencing (WES) as a cost-effective alternative to WGS, we extended our evaluation to 41 samples for which WES and WGS data were available (WES was generated as described earlier^20^). For 27 samples Constellation WES and WGA calls matched (data not shown). When comparing the variant sets produced by both methods, discrepant *CYP2D6* calls were likely due both to the lack of information for intronic variants, misaligned reads from pseudogenes, and the possible presence of false-positive WES variant calls. We are currently further exploring the utility of Constellation for pharmacogene diplotype calling from WES data as well as pharmacogene custom capture panels such as PGRNseq that have been developed by the Pharmacogenetics Research Network (www.pgrn.org).

Herein, the examination of analytic and clinical utility of Constellation was limited to *CYP2D6*. For clinical use, Constellation will need to impute function and/or activity scores in ~100 ADMER genes, together with return of results in the setting of electronic clinical decision support for associated drug, dose and exposure guidance for individual patients. Constellation is extensible to any polymorphic locus in which a comprehensive library of functionally relevant haplotypes and defining variant sets can be determined, and for which paired short reads align unambiguously. WGS technology continues to improve producing longer read lengths at high quality, further reducing ambiguous read mappings. For *CYP2D6,* the most polymorphic ADMER locus, the current complete diplotype set contained 7,140 entries. Similar complexity can be anticipated for the *CYP2A* locus, but the number of clinically relevant substrates is considerably less compared with *CYP2D6;* the remaining ~98 ADMER genes are considerably less complex. Although clinical validation for ~100 genes is onerous, *in silico* mapping may reduce that burden to a small subset of structural variations and gene—pseudogene instances where empiric evidence is needed.

In addition to pharmacogenomics, there is potential for the extension of Constellation to common complex diseases where actionable clinical results have been difficult to derive from whole-genome sequences. Despite abundant knowledge of genetic variants conferring risk, pathogenicity probability is often related to single nucleotide variation. By extending Constellation from the integration of intra-locus variation to include multiple loci, calculating a cumulative risk score for complex diseases in individual patients. Re-application of such methods to genome-wide association datasets could allow parameterisation of the scoring algorithm for individual common diseases. As technological advances continue to improve the speed and accuracy of WGS, while decreasing its cost, computational tools such as Constellation will increasingly become critical for translation of findings between domains such as genomics, genetics, clinical pharmacology and medicine, which will be essential for broad adoption of precision medicine.

## MATERIALS AND METHODS

### Subjects

This study was approved by the Institutional Review Board of Children’s Mercy—Kansas City. Informed written consent was obtained from adult subjects and parents of living children. DNA samples from consecutive subjects with sufficient amounts of DNA available for the study were chosen for analysis. Retrospective samples, UDT002 and UDT173, were from a validation set with known molecular diagnoses for genetic diseases.^19^ Probands were suspected of having a monogenetic disease, but without a definitive diagnosis at time of enrollment.^19^ HapMap subjects (NA12878, NA12877, NA12882, NA07019 and NA12753, NA18507 and NA19685 were obtained from the Coriell Institute for Medical Research, NJ) of which NA12878, NA12877 and NA12882 were a familial trio. NA18507 and NA19685 were chosen because they carry *CYP2D6* gene duplications. Subject ethnicity and relatedness are shown in Table 1.

### *In silico* modelling

In order to assess the ability to align short sequence reads uniquely to their correct location within the *CYP2D* locus (GRCh37, Chr22:42,518,000–42,555,000), simulated single and paired-end reads were generated from the *CYP2D6* reference sequence of the 37-kb target region and then mapped to the entire reference genome (note that the sequence in GRCh37 corresponds to CYP2D6*2). *CYP2D6* region reads were simulated with a quality score of 36, tiling interval of five nucleotides, and no mismatches from the reference genome, with sequence coverage of ×30 over the target region. Single reads were generated in lengths of 50, 100, 200, 350, 500, 1,000, 2,000, 3,000, 4,000 and 5,000 nucleotides. Paired-end reads were created with read lengths of 100, 125, 150, 200 and 350 nucleotides and with simulated sequencing library sizes of 500, 750 and 1,000 nucleotides for each read length. Each read set was aligned against the GRCh37.p5 reference genome using Genomic Short-read Nucleotide Alignment Program (GSNAP) allowing for multiple alignments. Reads which aligned uniquely to their exact position of origin were counted as mappable; reads with unique alignments to incorrect position were labelled as unmappable, and reads that aligned to multiple positions were labelled as ambiguous. Results were compiled for each read set to determine the minimum read size required to resolve the Chr22:42,518,000–42,555,000, with a specific focus on *CYP2D6*.

### *CYP2D6* genotype analysis

#### Allele nomenclature

*CYP2D6* haplotypes (commonly referred to as ‘star’ alleles) are designated by an asterisk and a combination of roman letters and Arabic numerals, as defined by the Human Cytochrome P450 (CYP) Allele Nomenclature Database at www.cypalleles.ki.se/.^3^ Of note, the *CYP2D6* haplotype in the reference genome (GRCh37) corresponds to CYP2D6*2 and not CYP2D6*1, which is regarded as the wild-type or reference sequence in the pharmacogenomic community.

#### Long range (XL-PCR)

*CYP2D6* genotyping was performed as described.^37,39–44^ Briefly, long-range PCR was used to amplify a 6.6-kb fragment encompassing the entire *CYP2D6* gene (fragment A), a 3.5-kb fragment from the intergenic region of *CYP2D6* duplication structures (fragment B) and a 5-kb fragment from *CYP2D7/2D6* hybrid structures (fragment H).^42^ Presence of fragments was determined by band visualisation following agarose gel electrophoresis. The gene regions amplified are shown in Supplementary Figure 1.

#### TaqMan genotyping

To test for single-nucleotide variations, XL-PCR amplicons were diluted 2,000-fold and used in TaqMan genotyping assays (Thermo Fisher Scientific, Waltham, MA, USA) to detect a panel of *CYP2D6* (NM_000106.5) sequence variations allowing us to assign haplotypes defined as CYP2D6*2, *3, *4, *6, *7, *9, *10, *11, *17, *29, *31, *35, *41, *42 and *45. In the absence of these variants, the haplotype assigned was CYP2D6*1. *CYP2D6* duplications/multiplications, the CYP2D6*5 gene deletion, *CYP2D7/2D6* hybrid arrangements (collated under the CYP2D6*13 designation^45^), and other *CYP2D6/2D7* hybrids (such as CYP2D6*68), were identified by a quantitative CNV assay and confirmed by long-range PCR.^37,44^ Furthermore, duplicated gene copies were genotyped by performing TaqMan genotyping assays on an XL-PCR product (fragment D) that encompasses the entire duplicated gene copy. An overview of the genotyping strategy and additional details are provided in Supplementary Table 1.

#### Activity score

An Activity Score (AS) was assigned to each allele as described previously^44^ with the traditional phenotype classifications poor, intermediate, extensive and ultrarapid metabolisers in accordance with guidelines from the Clinical Pharmacogenetics Implementation Consortium.^9–11^

#### Sanger sequencing

The *CYP2D6* locus, including at least 600 and 150 nucleotides upstream and downstream of the translation start and stop codons, respectively, was sequenced in both directions. The 6.6-kb *CYP2D6* fragment A (Supplementary Figure 1) was purified with a GenElute PCR Clean-up kit (Sigma, St Louis, MO, USA). Sequencing was performed with BigDye Terminator chemistry on a 3,730× DNA analyzer (Thermo Fisher Scientific, Waltham, MA, USA). Sequences were assembled using Sequencer software V4.9 (GeneCodes, Ann Arbor, MI, USA) and compared to the *CYP2D6* accessions M33388.1 and AY545216.^39^

To determine the haplotypes of two novel subvariants of known *CYP2D6* haplotypes in subject CMH396, allele-specific XL-PCR was performed with primer −740C>T to generate a 5.5-kb XL-PCR product from the CYP2D6*1 variant as described.^46^

#### Whole-genome sequencing

WGS was performed as previously described.^19^ Briefly, 1,000 ng of DNA was sheared to an average size of 350 nt using a Covaris S2 Biodisruptor, end repaired, A-tailed and adaptor ligated using Illumina TruSeq PCR free according to manufacuter’s protocol (Illumina Inc., San Diego, CA, USA). Quantitation was performed using real-time PCR. Samples for WGS were sequenced on HiSeq 2,500 instruments (Illumina, San Diego, CA, USA) on rapid run or high-throughput mode to a read depth of ~30× from ~100 GB of total data with 2×100 nt or 2×125 nt reads. Samples were aligned and variants called with GSNAP and the Genome Analysis Tool kit (GATK), respectively,^47–49^ relative to the GRCh37 CYP2D6*2 reference, yielding 5.1 million variants per genome as a variant call format (.vcf) file (Supplementary Table 2). Variants were called using methods previously described,^19^ briefly positions were downsampled to 750 reads using bases with sequence quality ≥20, mapping quality ≥17 and with a minimum phred-scaled confidence score of 20.0. Subsequently, variants were compared with the standard CYP2D6*1 reference (AY545216) allele.

### Constellation: data input and output

Data inputs for Constellation were .vcf files, a gene directory with chromosomal position, and a nomenclature file for each locus to be diplotyped. The position file contained the location of the gene transcript [Chr:start—stop] according to the GRCh37 reference. Bam file coverage was utilised to allow Constellation check for minimum coverage per gene (10× read coverage); if this threshold was not met, then the gene was flagged as uncallable and no diplotype was called. The nomenclature file contained the full set of possible genotypes, one per line, in the format [allele_name<tab> var1,var2,var3], with variants annotated as [Chr~start~stop~var]. The output is the most likely diplotype for that sample. Constellation was implemented in the Java programming language.

To determine copy-number variation a BAM file (.bam) and a BED file (.bed) were used. The BAM file contained aligned reads and the BED file contained a list of sentinel regions marked by position against the aligned reference. Local *CYP2D6* sentinel regions were evaluated for depth of coverage as were paired control regions. Significant deviation from expected ratios of coverage indicated the presence of a gene deletion (CYP2D6*5) or duplication.

## Figures and Tables

**Table 1:** Summary of diplotype and activity score assignments and phenotype predictions for different methods, the consensus reference and Constellation

*Subject ID*	*Related*	*Ethnicity*	*CYP2D6 gene copy number*	*CYP2D6 diplotypes*				*CYP2D6 Activity Score*		*Phenotype prediction*
				*TaqMan genotype*	*Sanger sequnecing*	*Consensus reference*	*Constellation*	*Consensus reference*	*Constellation*	
CMH 064	No	C	1	*35/*35	*35/*35	*5/*35	*5/*35	1	1	EM
CMH 076	No	AA	*2*	*2/*2	*2/*2 #	*2/*2	*2/*2	2	2	EM
CMH 172	No	Mex	*2*	*1/*1	*1/*1	*1/*1	*1/*1	2	2	EM
UDT 002	No	n/a	*2+6/7 hyb*	*4/*4	*4/*4 #	*4/*68+*4	*4/*4	0	0	PM
UDT 173	No	n/a	*2+6/7 hyb*	*1/*4	*1/*4 #	*1/*68+*4	*1/*4	1	1	EM
CMH 557	No	C	*2*	*1/*1	*ND*	*1/*1	*1/*1	2	2	EM
CMH 563	No	C	*2*	*1/*2	*ND*	*1/*2	*1/*2	2	2	EM
CMH 010	No	C	*2*	*1/*41	*ND*	*1/*41	*1/*41	1.5	1.5	EM
CMH 154	No	C	*2*	*1/*41	*ND*	*1/*41	*1/*41	1.5	1.5	EM
CMH 487	No	C	*2*	*1/*35	*ND*	*1/*35	*1/*35	2	2	EM
CMH 545	No	C	*2*	*1/*4	*ND*	*1/*4	*1/*4	1	1	EM
CMH 589	No	C	*2*	*4/*4	*4/*4 #	*4/*4	*4/*4	0	0	PM
CMH 663	No	C	*2*	*4/*41	*ND*	*4/*41	*4/*41	0.5	0.5	IM
CMH 677	No	C	*2*	*4/*4	*ND*	*4/*4	*4/*4	0	0	PM
CMH 731	No	C	*2*	*4/*10	*4/*10	*4/*10	*[mac]*	**0.5**	**No call**	IM
NA07019	No	C	*2*	*1/*4	*ND*	*1/*4	*1/*4	1	1	EM
NA12753	No	C	*2*	*2/*3	*ND*	*2/*3	*2/*3	1	1	EM
NA19685	No	Mex	*3*	*1/*2	*ND*	*1/*2x2	*1/*2 (+)	3	3	UM
NA18507	No	Yoruban	*3*	*2/*4	*2/*4	*2/*4x2	*[mac](+)*	**1**	**No call**	EM
CMH 186	M	Mex	*2+6/7 hyb*	*2/*4	*2/*4 #	*2/*68+*4	*2/*4	1	1	EM
CMH 202	F	Mex	*2*	*4/*45 or 46	*4/*45	*4/*45	*4/*45	1	1	EM
CMH 184	C-1	Mex	*2*	*2/*4	*2/*4	*2/*4	*2/*4	1	1	EM
CMH 185	C-2	Mex	*2+6/7 hyb*	*4/*4	*4/*4 #	*4/*68+*4	*4/*4	0	0	PM
CMH 224	M	n/a	*2*	*4/*41	*4/*41	*4/*41	*4/*41	0.5	0.5	IM
CMH 222	C-1	n/a	*2*	[*2]/*4	*4/*59 #	*4/*59	*4/*59	0.5	0.5	IM
CMH 223	C-2	n/a	*2*	*1/*41	*33/*41	*33/*41	*33/41	1.5	1.5	EM
CMH 248	M	C	*2*	*1/*41	*1A/*41	*1/*41	*1/*41	1.5	1.5	EM
CMH 249	F	C	*2*	*4/*35	*4/*35	*4/*35	*4/*35	1	1	EM
CMH 446	C-1	C	*2*	*1/*35	*1A/*35	*1/*35	*1/*35	2	2	EM
CMH 447	C-2	C	*2*	*35/*41	*35A/*41	*35/*41	*35/*41	1.5	1.5	EM
CMH 397	M	AA/AI	*2*	*17/*45	*17/*45 #	*17/*45	*17/*45	1.5	1.5	EM
CMH 398	F	AA/AI	*2*	*1/*17	*1/*17 #	*1/*17	*1/*17	1.5	1.5	EM
CMH 396	C	AA/AI	*2*	*1/*17	*1/*17 #	*1/*17	*1/*17	1.5	1.5	EM
CMH 437	M	AA	*2*	*1/*41	*1/*41 #	*1/*41	*1/41	1.5	1.5	EM
CMH 438	F	AA	*2*	*1/*17	*1/*17 #	*1/*17	*1/*17	1.5	1.5	EM
CMH 436	C	AA	*2*	*1/*1	*1/*1 #	*1/*1	*1/*1	2	2	EM
CMH 570	M	C	*2*	*1/*1	*1/*1	*1/*1	*39/*95	**2**	**Unknown**	EM
CMH 571	F	C	*2*	*1/*4	*4/*33	*4/*33	*4/*33	1	1	EM
CMH 569	C	C	*2*	*1/*4	*ND*	*1/*4	*1/*4	1	1	EM
CMH 579	M	C	*2*	*1/*2	*ND*	*1/*2	*1/*2	2	2	EM
CMH 580	F	C	*2*	*2/*41	*ND*	*2/*41	*2/*41	1.5	1.5	EM
CMH 578	C	C	*2*	*1/*2	*ND*	*1/*2	*1/*2	2	2	EM
CMH 630	M	n/a	*2*	*1/*2	*ND*	*1/*2	*1/*2	2	2	EM
CMH 631	F	n/a	*2*	*2/*17	*17/*84 #	*17/*84	*17/*84	Unknown	Unknown	Unknown
CMH 629	C	MR	*2*	*1/*17	*ND*	*1/*17	*1/*17	1.5	1.5	EM
CMH 673	M	C	*2*	*1/*35	*1/*35	*1/*35	*35/*83	2	1	EM
CMH 674	F	C	*1*	*2/*2	*ND*	*2/*5	*2/*5	1	1	EM
CMH 672	C	C	*1*	*1/*1	*ND*	*1/*5	*1/*5	1	1	EM
CMH 681	M	C	*2*	*1/*4	*1/*4 #	*1/*4	*1/*4	1	1	EM
CMH 682	F	C	*2*	*2/*2	*ND*	*2/*2	*2/*2	2	2	EM
CMH 680	C	C	*2*	*1/*2	*ND*	*1/*2	*1/*2	2	2	EM
CMH 729	M	C	*2*	*1/*41	*1/*41 #	*1/*41	*1/*41	1.5	1.5	EM
CMH 730	F	C	*1*	*2/*2	*59/*59	*5/*59	*5/*59	0.5	0.5	IM
CMH 728	C	C	*1*	*1/*1	*1/*5 #	*1/*5	*1/*5	1	1	EM
CMH 679	M	C	*2*	*4/*4	*ND*	*4/*4	*4/*4	0	0	PM
CMH 678	C	C	*2*	*1/*4	*ND*	*1/*4	*1/*4	1	1	EM
CMH 719	M	C	*2*	*1/*2	*ND*	*1/*2	*1/*2	2	2	EM
CMH 718	C	C	*2*	*1/*2	*ND*	*1/*2	*1/*2	2	2	EM
NA12878	M	Eur	*2+6/7 hyb*	*3/*4	*3/*4	*3/*68+*4	*3/*4	0	0	PM
NA12877	F	Eur	*2+6/7 hyb*	*3/*4	*4/*4	*4/*68+*4	*4/*4	0	0	PM
NA12882	C	Eur	*2+6/7 hyb*	*3/*4	*4/*4	*4/*68+*4	*4/*4	0	0	PM

Abbreviations: AA, African American; AI, American Indian; C, Caucasian; Ch, child; Ch-1, child 1; Ch-2, child 2; CNV, copy-number variation; EM, extensive metaboliser phenotype; Eur, European Ethnicities; F, father; IM, intermediate metaboliser phenotype; M, mother; MR, mixed race; No, not related; PM, poor metaboliser phenotype; UM, ultrarapid metaboliser phenotype; WGS, whole-genome sequencing.

TaqMan refers to genotype analysis using a panel of genotyping assays (see Supplementary Table 1). CNV refers to quantitative multiplex PCR that determines *CYP2D6* gene copy number (deletion, duplication, multiplication and gene hybrids). This assay was complemented by genotyping XL-PCR amplicons generated from duplicated or hybrid gene copies (Supplementary Figure 1) or sequencing. The number of gene copies are as indicated; the presence of *CYP2D6/CYP2D7* gene hybrids (6/7 hyb) are also shown. Sanger refers to diplotype calls based on Sanger sequencing of a 6.6-kb long XL-PCR product encompassing the *CYP2D6* gene (Supplementary Figure 1). Consensus reference indicates calls derived from a combination of CNV, TaqMan and Sanger sequencing. Constellation refers to calls made by the Constellation software using .vcf files generated from WGS. Activity Scores (AS) were assigned to diplotypes derived from the consensus reference diplotypes and Constellation. Inconsistent calls between the consensus reference calls and Constellation are bolded. Phenotype prediction is consistent between the consensus reference and Constellation calls with the exception of three cases. (+) denotes that the subject was identified as having a duplication. [mac], multiple ambiguous calls causing a ‘no call’ result. #, novel subvariant(s) identified (see Supplementary Figure 3 for details). For brevity, this is only annotated in the column labelled ‘Sanger’. [*2], TaqMan genotype result for SNP rs16947 was not conclusive. Allele subtype assignments are not shown in this table, but provided for each individual in Supplementary Figure 3. Subjects with a CMH or UDT-prefix are patient samples, those with a NA-prefix were obtained from the Coriell Institute. Relatedness of subjects is as indicated. Coriell samples are annotated as European (Eur) in the Coriell database.
