# Constellation: a tool for rapid, automated phenotype assignment of a highly polymorphic pharmacogene, *CYP2D6*, from whole-genome sequences

**DOI:** 10.1038/npjgenmed.2016.39

**Published:** 2017-01-11

**Authors:** Greyson P Twist, Andrea Gaedigk, Neil A Miller, Emily G Farrow, Laurel K Willig, Darrell L Dinwiddie, Josh E Petrikin, Sarah E Soden, Suzanne Herd, Margaret Gibson, Julie A Cakici, Amanda K Riffel, J Steven Leeder, Deendayal Dinakarpandian, Stephen F Kingsmore

**Correction to**: *npj Genomic Medicine* (2016) **1**, 15007; doi:10.1038/npjgenmed.2015.7; published online 13 January 2016

The tool reported in this article with the name ‘Constellation’, which allows rapid, automated phenotype assignment of a highly polymorphic pharmacogene, CYP2D6, from whole-genome sequences, has been renamed ‘Astrolabe’ to avoid conflict and confusion with another software tool. Astrolabe is available at no cost for academic research users and can be licensed for commercial use. Astrolabe can be downloaded at https://www.childrensmercy.org/genomesoftwareportal/. Registration is required.

