# Supplementary material for: Constellation: a tool for rapid, automated phenotype assignment of a highly polymorphic pharmacogene, CYP2D6, from whole-genome sequences
Source: NPJ Genom Med. 2016 Jan 13;1:15007–. doi: 10.1038/npjgenmed.2015.7 (PMC5685293; doi:10.1038/npjgenmed.2015.7)
Supplement: Supplementary Information [file npjgenmed20157-s1.doc]

**Supplementary Information**

**Suppl Figure 1**

**Long-range PCR products used to characterize the** CYP2D6**gene locus**. This graph only shows the structural variants found in the current study.

Panel **A** shows the CYP2D6 reference locus. XL-PCR fragment A amplifies the *CYP2D6* gene or variant alleles as shown for the *CYP2D6*2* example; this fragment was subsequently used for genotyping and Sanger sequencing. Panel **B** displays the CYP2D6*5 gene deletion allele. The forward XL-PCR primer is located downstream of *CYP2D7* and the reverse primer binds downstream of *CYP2D6*. In the presence of a *CYP2D6* deletion the two primer binding sites are close enough to support fragment formation. Panel **C** represents duplication arrangements; fragment A is amplified from both gene copies. Fragment B targets the intergenic region and only generates an amplicon if a duplication structure features a *CYP2D6*-derived downstream region. Fragment D amplifies the duplicated gene copy only; this fragment was used to genotype the duplicated gene copy. Panel **D shows the** *CYP2D6*68+*4* tandem. The **68* gene copy is a *CYP2D6/2D7* hybrid. Due to the presence of a *CYP2D7*-derived downstream region, fragment B does not amplify. Fragment D, however does amplify because both primer binding sites correspond to *CYP2D6*. The *CYP2D6*68* hybrid can also be generated with a *CYP2D6*-specific forward and a *CYP2D7*-specific reverse primer as indicated.

CYP2D6, CYP2D7, and CYP2D8 genes are shown in white, red, and dark gray boxes, respectively. The 600 bp repeat element immediately downstream of *CYP2D6* and *CYP2D7* exon 9 is shown in blue. Alu repetitive elements (REP) are in red and light gray; REP-del indicates a fused repeat element generated by a large deletion involving parts of those elements from both genes. PCR fragments generated are represented as lines and fragment lengths provided in kilo base pairs (kb).

**Suppl Figure 2**

*In silico* modeling of the uniqueness of alignments of simulated short-read sequences to the region of Chromosome 22 containing *CYP2D6 andCYP2D7*.

7401 simulated singleton reads of 50 to 5000 nt (**a**) and 7401 paired-end reads of 100 to 350 nt (**b**) in length were generated from this region. For paired-end reads, insert lengths were varied from 300 to 800 nt. Exons, introns and repetitive genomic segments to which reads mapped uniquely with GSNAP are shown in green; Regions to which reads did not map uniquely are shown in red.

**Suppl Figure 3**

Summary of genotyping, Sanger and Whole Genome Sequencing results.

Rows 2-9 provide rs numbers, SNP coordinates for genome reference hg19 and coordinates for the commonly used *CYP2D6* reference sequences M33388 and AY545216. The Human Cytochrome P450 Nomenclature Data Base ([www.cypalleles.ki.se/cyp2d6.htm](../../../../%5C%5Ccmh%5Cgroups%5CGenome%20Center%5CTeam%20Folders%5CCYP2D6%5CConstellation_paper%5CSubmitted%20files%5Cwww.cypalleles.ki.se%5Ccyp2d6.htm)) maps sequence variation on M33388, however, this sequence differs from AY545216 in three locations as indicated in row 3. The hg19 sequence (row 12) corresponds to *CYP2D6*2.* SNPs interrogated by TaqMan genotype assays are shown in row 13 and are highlighted in purple. Gray text SNV annotations indicate that these were identified by WGS variation calls, but not observed in any subject(s) for which Sanger sequencing was performed; these are likely due to unspecific read alignments. Row 381 denotes SNV locations in 5’ and 3’ flanking regions, exons, and introns; amino acid changes and other functional consequences are provided in row 383. As shown in row 385, white boxes indicate that a nucleotide(s) at that position corresponds to *CYP2D6*1* (AY545216 reference, row 11), gray boxes indicates heterozygosity and black boxes denote homozygous variant. SNPs defining novel haplotypes are highlighted in blue (light blue, heterozygous; dark blue homozygous). Yellow boxes denote the regions not covered by Sanger sequencing. For each individual three rows summarize variant calls identified by the WGS GSNAP-GATK caller, Sanger sequencing and by TaqMan genotyping (i.e. rows 15-17 for subject CMH064) and two rows indicate on which allele SNVs are located (haplotype) (i.e. rows 18 and 19 for subject CMH064). Respective genotype and allele assignments are given in column CJ. For example, Constellation determined a *CYP2D6*5/35A* diplotype using WGS GSNAP-GATK variant call input data. Sanger sequencing and TaqMan genotyping each determined *CYP2D6*35/*35* which together with CNV determination of 1 gene copy constitutes the consensus reference *CYP2D6*5/*35* genotype. Relatedness, ethnicity and subject IDs are provided in columns CK-CO.

**Suppl Table 1**

This table summarizes which SNPs were genotyped, their respective rs ID numbers, nucleotide changes, positions on commonly used reference sequences, on which allele they occur and IDs of TaqMan assays for their detection.

There are no rs number available for one SNP and structural variants as indicated by ‘-‘. Some SNPs are part of multiple allele definitions (haplotypes) and may occur on alleles not shown here. The table lists only those alleles subjects were genotyped for. X denotes that assay was performed. ‘-‘ denotes that assay was not performed. Position coordinates are shown for two common *CYP2D6*1* reference sequences. M33388 is used by the nomenclature committee; AY545216 differs from M33388 in four positions shifting coordinates. One SNP was genotyped by a restriction fragment length polymorphism (RFLP) assay as described by Gaedigk et al (PMID 15768052). Gene deletion, duplication and *CYP2D6/2D7* and *CYP2D7/2D6* hybrid structures were interrogated by a quantitative copy number variation (qCNV) assay^26^ and confirmed by XL-PCR^25^.

**Suppl Table 2**

WGS metrics for 61 samples. GB, gigabases; ACMG, American College of Medical Genetics pathogenicity classification of nucleotide variants; Q, Phred-like quality score.

**Suppl Table 3**

Summary of the 119 haplotype definitions included into the current version of Constellation. Alleles are as defined by the Human Cytochrome P450 Nomenclature database at [www.cypalleles.ki.se](http://www.cypalleles.ki.se/). As indicated, four haplotypes, *CYP2D6*17, *35, *41* and **59*, received an additional definition based on unpublished sequence data that included all SNPs present on respective alleles. Activity of alleles was assigned as normal (Activity Score = 1), decreased (Activity Score = 0.5) or no activity (Activity Score = 0). Haplotypes for which in vivo activity is unknown or uncertain (e.g. only in vitro data are available) have no assigned activity.

**Suppl Table 4**

Summary of sequence variations found in the suballeles not defined on the Nomenclature data base.

**Supplementary Results**

**Inconsistencies between TaqMan genotyping and sequence information**

Sanger sequencing (fragment A encompassing *CYP2D6*, Suppl Fig1) and WGS revealed the presence of sequence variations that were not detected by TaqMan genotyping assays. The following paragraph briefly describes these subjects.

CMH223 and CMH571

A SNP at position 2483G>T was identified in both subjects by Sanger sequencing changing allele assignments from *CYP2D6*1* to **33.* Constellation correctly called this allele as *CYP2D6*33*. Notably, this allele carries additional sequence variations that are not annotated on the CYP2D6 Nomenclature Database.

CMH222 and CMH 730

2291G>A and 2939G>A were found by Sanger sequencing revising initial *CYP2D6*2* assignments to *CYP2D6*59.* The revision to *CYP2D6*59* is functionally relevant, since the *in vitro* activity of *CY2D6*2* is normal, whereas that of *CY2D6*59* is decreased^50^. Constellation initially did not call *CYP2D6*59* based on the allele definition provided by the *CYP2D6* Nomenclature Database. Updating Constellation with a complete set of nucleotide variants from Sanger sequencing enabled the algorithm to call *CYP2D6*59* in both subjects.

Of note, one of the key SNPs defining on *CYP2D6*59*(2939G>A) is interfering with the TaqMan genotype assay detecting rs16947 (2850C>T present on **2* and many other haplotypes)^31^. Therefore, the TaqMan *CYP2D6*2* assignment was tentative and hence is shown in brackets in Table 1.

CMH631

Sanger sequencing identified a complement set of SNPs indicating the presence of *CYP2D6*84*. Since all SNVs of *CYP2D6*84* are annotated on the *CYP2D6* Nomenclature Database, Constellation did not require any revisions in order to call this allele correctly.

Novel allelic subvariants

Sequencing identified 15 SNPs (highlighted in blue in Suppl Fig 3) defining 12 suballeles that are not in the *CYP2D6* Nomenclature database (n=5 for **1*; n=2 for **2*; n= 4 for **4* and n=1 for **17*). Six of the suballeles were observed in only a single individual and six in at least two subjects. Of the 15 SNPs, 2760T>A was previously described in the *CYP2D6* Nomenclature database under the section ‘without haplotype information’. Another SNP, 2602G>T has previously been described as a *CYP2D6*17*var^34^, but was not designated as a suballele. There was also a novel suballele of *CYP2D6*17* that was defined by the lack of three SNPs (designated *CYP2D6*17 var2*). No designations will be assigned to these subvariants because none of the SNPs are nonsynonymous and will likely not change function.

SNPs constituting novel *CYP2D6* subvariants.SNP positions are according to M33388; Genome coordinates and other information are detailed in Suppl Fig 3 and Suppl Table 4.

**Refinements of haplotype definitions**

Using the allele definitions per the Human P450 Nomenclature Data Base at [www.cypalleles.ki.se/](../../../../%5C%5Ccmh%5Cgroups%5CGenome%20Center%5CTeam%20Folders%5CCYP2D6%5CConstellation_paper%5CSubmitted%20files%5CRevision%5Cwww.cypalleles.ki.se%5C), Constellation consistently miscalled a number of alleles among them *CYP2D6*4.* For this particular allele incomplete allele definitions were suspected as the underlying cause, i.e. allele definitions are based on exonic sequences only (any SNPs in introns and flanking regions, if there are any, are not listed). Therefore, *CYP2D6*4* sub allele definitions (**4A - *4E*) were removed from the allele definition set. For other miscalled alleles, among them *CYP2D6*35*, the allele definition file was updated based on fully sequenced haplotypes (using existing data from other investigations). For instance, the complete sequence for *CYP2D6*35* contained the intron 1 conversion (a short *CYP2D7*-derived sequence) which is currently not annotated on the Nomenclature web page. By improving allele definitions and respective Constellation input files, we achieved markedly higher match scores, which in turn resulted in an overall improvement of allele calls. As described above, Constellation was also only able to accurately call *CYP2D6*59* after its definition included all variants.

This emphasizes the importance of complete haplotype definitions that include exon, intron and flanking gene regions for optimal performance of Constellation (and other algorithms that are based on haplotype definitions.).
