# Supplementary material for: Constellation: a tool for rapid, automated phenotype assignment of a highly polymorphic pharmacogene, CYP2D6, from whole-genome sequences
Source: NPJ Genom Med. 2016 Jan 13;1:15007–. doi: 10.1038/npjgenmed.2015.7 (PMC5685293; doi:10.1038/npjgenmed.2015.7)

Suppl Figure 1

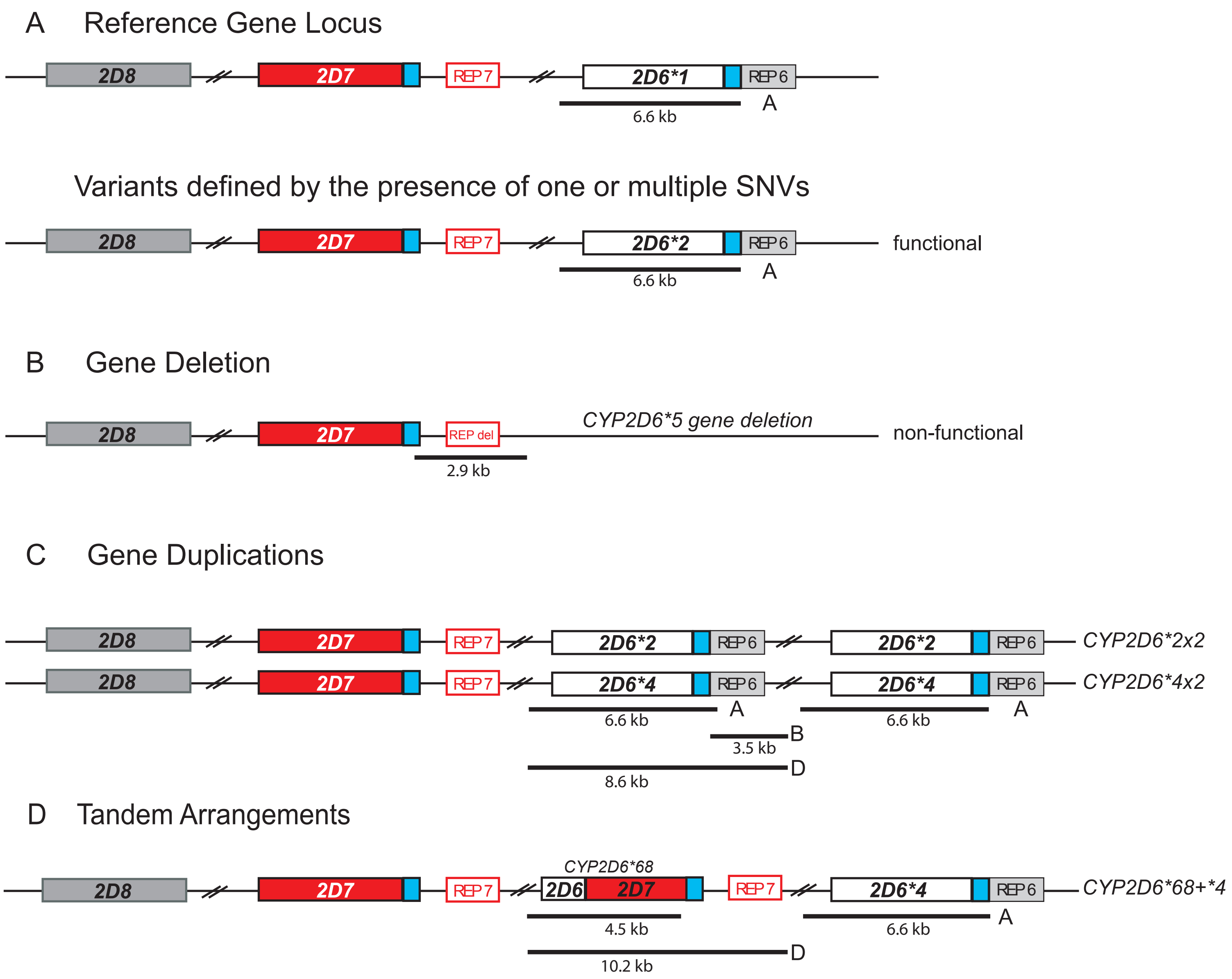

Supplement: Supplementary Figure 1 [file npjgenmed20157-s2.pdf]
