# Supplementary material for: Constellation: a tool for rapid, automated phenotype assignment of a highly polymorphic pharmacogene, CYP2D6, from whole-genome sequences
Source: NPJ Genom Med. 2016 Jan 13;1:15007–. doi: 10.1038/npjgenmed.2015.7 (PMC5685293; doi:10.1038/npjgenmed.2015.7)

A

| Read Length | Correctly Aligned | Miss-aligned | Multi align | Total | % Correctly Aligned | % Multi-Aligned |
|---|---|---|---|---|---|---|
| 50 | 5160 | 0 | 2241 | 7401 | 69.72 | 30.28 |
| 100 | 5906 | 0 | 1495 | 7401 | 79.80 | 20.20 |
| 200 | 6317 | 0 | 1084 | 7401 | 85.35 | 14.65 |
| 350 | 6509 | 0 | 892 | 7401 | 87.95 | 12.05 |
| 500 | 6629 | 0 | 772 | 7401 | 89.57 | 10.43 |
| 1000 | 6889 | 0 | 512 | 7401 | 93.08 | 6.92 |
| 2000 | 7289 | 0 | 112 | 7401 | 98.49 | 1.51 |
| 3000 | 7401 | 0 | 0 | 7401 | 100.00 | 0.00 |
| 4000 | 7401 | 0 | 0 | 7401 | 100.00 | 0.00 |
| 5000 | 7401 | 0 | 0 | 7401 | 100.00 | 0.00 |

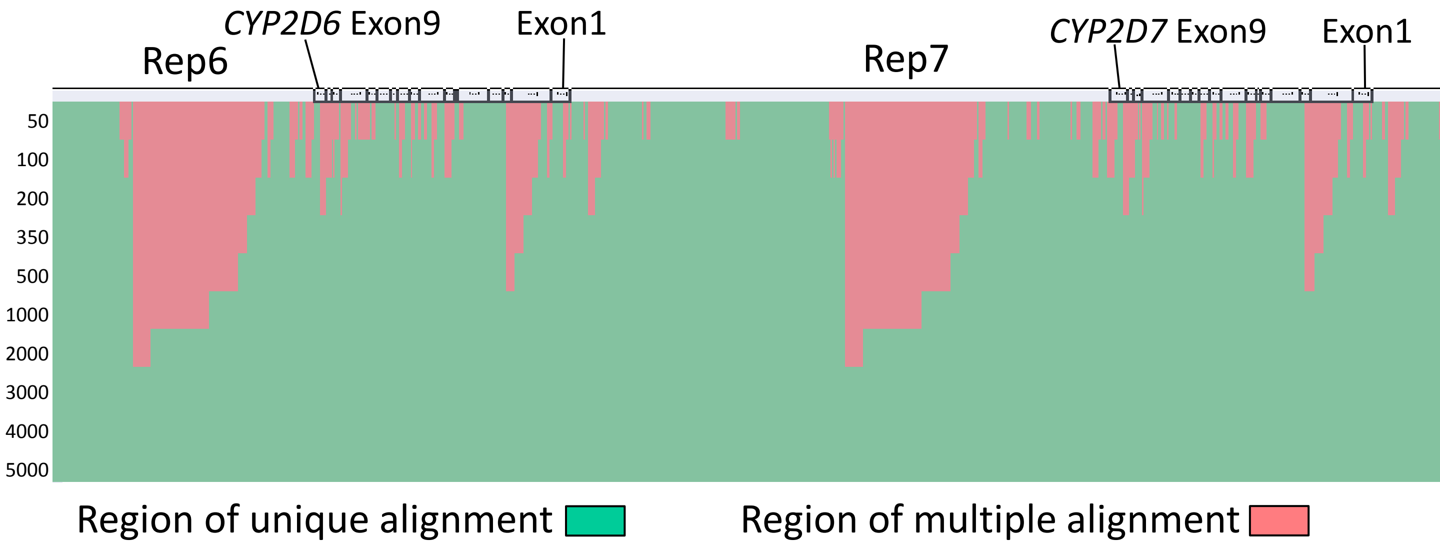


B

| Simulation | Read Length | Insert Length | Total Size | total reads | Total # of pairs | Correctly Aligned | Did not align | Multi Aligned |
|---|---|---|---|---|---|---|---|---|
| A | 100 | 300 | 500 | 14802 | 7401 | 6576 | 0 | 825 |
| B | 100 | 550 | 750 | 14802 | 7401 | 6687 | 0 | 714 |
| C | 100 | 800 | 1000 | 14802 | 7401 | 6832 | 0 | 569 |
| D | 125 | 250 | 500 | 14802 | 7401 | 6576 | 0 | 825 |
| E | 125 | 500 | 750 | 14802 | 7401 | 6724 | 0 | 677 |
| F | 125 | 750 | 1000 | 14802 | 7401 | 6864 | 0 | 537 |
| G | 150 | 200 | 500 | 14802 | 7401 | 6595 | 0 | 806 |
| H | 150 | 450 | 750 | 14802 | 7401 | 6759 | 0 | 642 |
| I | 150 | 700 | 1000 | 14802 | 7401 | 6886 | 0 | 515 |
| J | 200 | 100 | 500 | 14802 | 7401 | 6627 | 0 | 774 |
| K | 200 | 350 | 750 | 14802 | 7401 | 6789 | 0 | 612 |
| L | 200 | 600 | 1000 | 14802 | 7401 | 6889 | 0 | 512 |
| M | 350 | 300 | 1000 | 14802 | 7401 | 6889 | 0 | 512 |

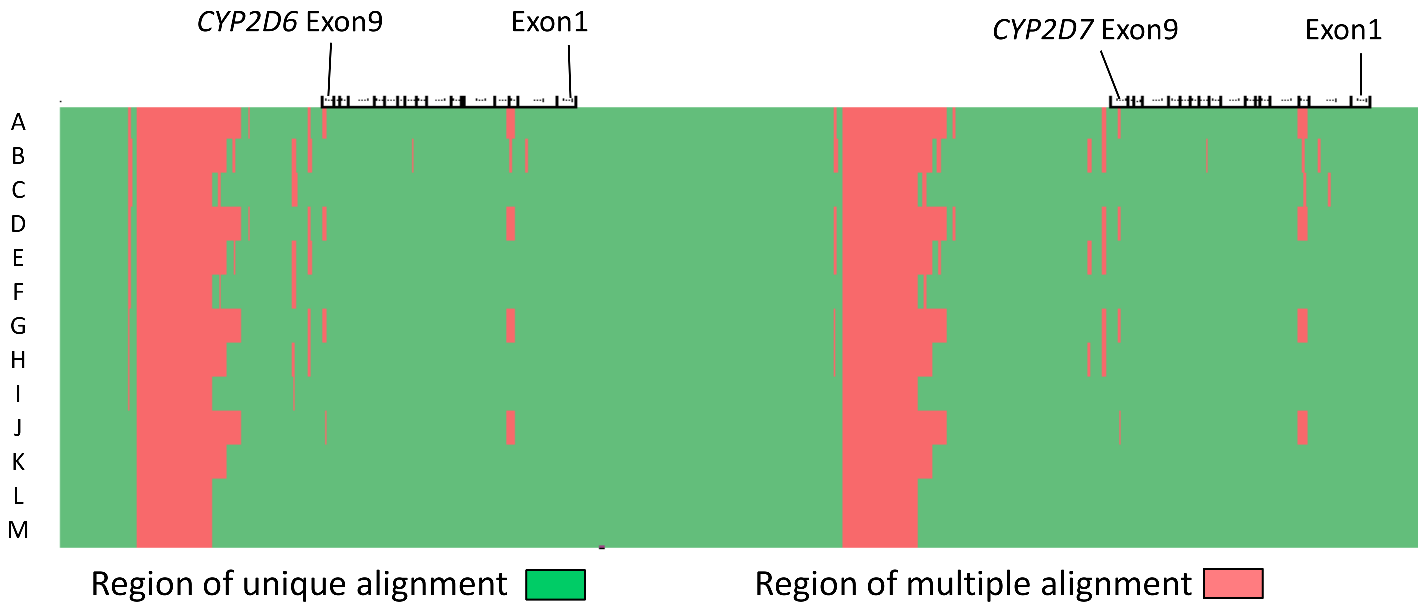

Supplement: Supplementary Figure 2 [file npjgenmed20157-s3.pdf]
